# Combined Curcumin and Lansoprazole-Loaded Bioactive Solid Self-Nanoemulsifying Drug Delivery Systems (Bio-SSNEDDS)

**DOI:** 10.3390/pharmaceutics14010002

**Published:** 2021-12-21

**Authors:** Abdulrahman Alshadidi, Ahmad Abdul-Wahhab Shahba, Ibrahim Sales, Md Abdur Rashid, Mohsin Kazi

**Affiliations:** 1 Department of Pharmaceutics, College of Pharmacy, King Saud University, Riyadh 11451, Riyadh Province, Saudi Arabia; abdu20141@hotmail.com; 2 Department of Clinical Pharmacy, College of Pharmacy, King Saud University, Riyadh 11451, Riyadh Province, Saudi Arabia; isales@ksu.edu.sa; 3 Department of Pharmaceutics, College of Pharmacy, King Khalid University, Abha 62529, Aseer, Saudi Arabia; mdrashid@kku.edu.sa; 4 Kayyali Chair for Pharmaceutical Industries, Department of Pharmaceutics, College of Pharmacy, King Saud University, Riyadh 11451, Riyadh Province, Saudi Arabia

**Keywords:** curcumin, lansoprazole, self-nanoemulsifying drug delivery systems (SNEDDS), solidification technique, capsule-in-capsule technology

## Abstract

Background: The current study aimed to design a novel combination of lansoprazole (LNS) and curcumin (CUR) solid oral dosage form using bioactive self-nanoemulsifying drug delivery systems (Bio-SSNEDDS). Methods: Liquid SNEDDS were prepared using the lipid-excipients: Imwitor988 (cosurfactant), Kolliphor El (surfactant), the bioactive black seed (BSO) and/or zanthoxylum rhetsa seed oils (ZRO). Liquid SNEDDS were loaded with CUR and LNS, then solidified using commercially available (uncured) and processed (cured) Neusilin^®^ US2 (NUS2) adsorbent. A novel UHPLC method was validated to simultaneously quantify CUR and LNS in lipid-based formulations. The liquid SNEDDS were characterized in terms of self-emulsification, droplet size and zeta-potential measurements. The solidified SNEDDS were characterized by differential scanning calorimetry (DSC), X-ray powder diffraction (XRD), scanning electron microscopy (SEM), in vitro dissolution and stability in accelerated storage conditions. Results: Liquid SNEDDS containing BSO produced a transparent appearance and ultra-fine droplet size (14 nm) upon aqueous dilution. The solidified SNEDDS using cured and uncured NUS2 showed complete solidification with no particle agglomeration. DSC and XRD confirmed the conversion of crystalline CUR and LNS to the amorphous form in all solid SNEDDS samples. SEM images showed that CUR/LNS-SNEDDS were relatively spherical and regular in shape. The optimized solid SNEDDS showed higher percent of cumulative release as compared to the pure drugs. Curing NUS2 with 10% PVP led to significant enhancement of CUR and LNS dissolution efficiencies (up to 1.82- and 2.75-fold, respectively) compared to uncured NUS2-based solid SNEDDS. These findings could be attributed to the significant (50%) reduction in the micropore area% in cured NUS2 which reflects blocking very small pores allowing more space for the self-emulsification process to take place in the larger-size pores. Solid SNEDDS showed significant enhancement of liquid SNEDDS stability after 6 months storage in accelerated conditions. Conclusions: The developed Bio-SSNEDDS of CUR and LNS using processed NUS2 could be used as a potential combination therapy to improve the treatment of peptic ulcers.

## 1. Introduction

Peptic ulcer disease (PUD), an ulceration of stomach and/or duodenum, can be defined as a mucosal breach that extends through the muscularis mucosa into the submucosa or even deeper layers. Common causes include *Helicobacter pylori* (*H. pylori*) infection and non-steroidal anti-inflammatory drugs (NSAIDs) [1]. According to World Health Organization (WHO), there were 87.4 million new cases of PUD in 2015 resulting in 267,500 deaths [2]. The global annual incidence of PUD ranges from 57.75 to as high as 141.9 per 100,000 person [3]. 

Gastroesophageal reflux disease (GERD) is likewise a common medical condition defined as “symptoms or complications resulting from refluxed stomach contents into the esophagus or beyond, into the oral cavity (including the larynx) or lung [4]. The global prevalence ranges from as low as 2.5% in East Asia to as high as 33.1% in the Middle East [4,5]. 

**Figure 1:**
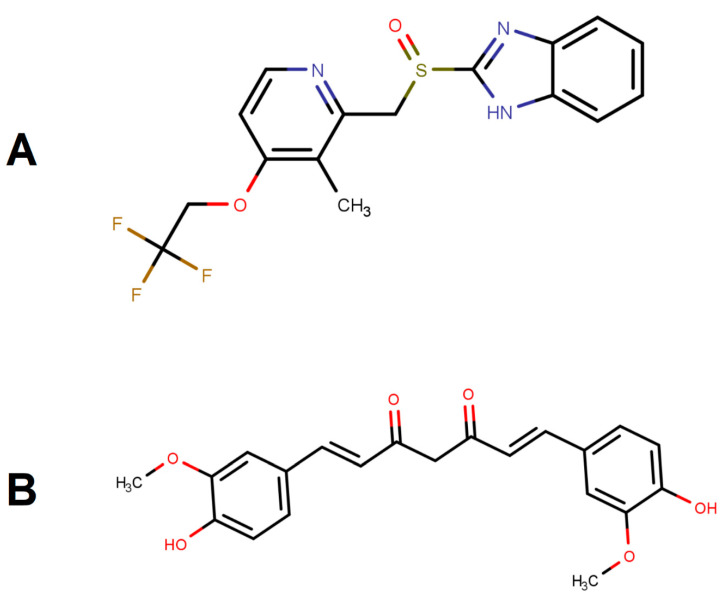
Chemical structure of (**A**) lansoprazole (MW: 369.4 g/mol) and (**B**) curcumin (MW: 368.4 g/mol). MarvinSketch was used for drawing, displaying and characterizing chemical structures, MarvinSketch 21.18.0, ChemAxon (https://www.chemaxon.com (accessed on 6 December 2021)).

Lansoprazole, (LNS, Figure 1A), a proton pump inhibitor (PPI), is a commonly used drug indicated for GERD, PUD, NSAID-associated gastric ulcers and hypersecretory conditions [6]. PPIs inhibit gastric acid secretion from active proton pumps by binding to the H+/K+-adenosine triphosphatase (ATPase) enzyme [7]. LNS is a lipophilic weak base (log *p* = 2.82), acid labile compound that belongs to the Biopharmaceutics Classification System (BCS) II (low solubility/high permeability). Such drugs usually experience poor oral bioavailability which is mainly due to its poor dissolution. Adequate formulation strategies must be applied to enhance aqueous solubility, dissolution and hence oral bioavailability of BCS II drugs [8]. 

Curcumin (CUR, Figure 1B) is a lipophilic bioactive substance, extracted from the rhizomes of the herb *Curcuma longa* L., having a wide variety of biological and pharmacological effects, such as anti-inflammatory [9], antioxidant [10,11], anti-depressive [12,13], memory improvement [14], antitumor [15,16], and hepatoprotective properties [17,18]. CUR is believed to exert its beneficial properties in various gastrointestinal disorders through various mechanisms by inhibiting gastric acid secretion and suppressing the pro-inflammatory levels of inducible nitric oxide synthase (iNOS) and tumor necrosis factor-alpha (TNF-alpha) in gastric ulcers [19]. Furthermore, its antioxidant activity, inhibition of NF-K-B activation, and inhibition of all branches of the arachidonic acid cascade (anti-inflammatory activity) have been proposed as the mechanism behind its effectiveness in GERD (Mahattanadul, Radenahmad et al., 2006). CUR, is a poorly water soluble compound with partition coefficient value of log *p* ≈ 3 [20].

When compared to LNS for the treatment of GERD in an in vivo study, CUR was effective in the treatment of GERD, although not as potent [21]. Further studies have confirmed its effectiveness in PUD [19] along with evidence from clinical trials on Curcuma longa Linn [22,23]. Therefore, it was hypothesized that a CUR/LNS formulation may be more effective than each agent alone and may be useful in reducing the incidence of treatment failures such as refractory GERD [24].

Self-nanoemulsifying drug delivery systems (SNEDDS) using bioactive lipid compounds have recently attracted attention as delivery systems to provide synergistic benefits and improve the oral bioavailability and/or biological activity of lipophilic drugs [25,26,27,28]. The demand for natural formulation active/inactive ingredients has emerged due to the increased risk of side effects posed by the synthetic compounds. Pharmaceutical ingredients from plant origin are generally safer as they produce less toxic metabolites.

Black seed oil (BSO) and Zanthoxylum rhetsa seed oil (ZRO) are valuable bioactive oils with several reported biological and pharmacological activities [26]. In particular, Rich et al. reported a significant improvement in symptoms for patients with chronic or recurrent functional dyspepsia taking a combination capsule of BSO and peppermint [29]. Mohtashami et al. reported a significantly lower Hong Kong index of dyspepsia severity scores and rates of *H. pylori* infection in BSO/honey based-formulation compared to placebo in patients with functional dyspepsia [30].

Currently no combination therapy is available either in liquid or solid dosage forms using CUR and LNS in SNEDDS formulations of bioactive lipid compounds. Therefore, in this study, an attempt was made to solidify liquid Bio-SNEDDS with cured and uncured Neusilin grade US2 to improve the solubility, dissolution and stability, of CUR/LNS combined oral formulation using bioactive oils. 

## 2. Materials and Methods

### 2.1. Plant Material and Extraction of Bioactive Oils

The methods of collection, extraction, and isolation of black seed oil and Zanthoxylum rhetsa seed oil (ZRO) were mentioned in detail in our previous publication [26].

### 2.2. Chemical and Reagents

Lansoprazole (LNS, purity = 99%) and curcumin (CUR, purity = 99.5%) were purchased from Enzo life Sciences, (Lausen, Switzerland). Imwitor 988 (medium chain mono and diglycerides C8–10), Kolliphor EL (KrEL), and polyvinylpyrrolidone (PVP-K30) were purchased from BASF, (Ludwigshafen, Germany). Hydrogenated castor oil (HCO30) was purchased from Nicole chemical co., (Tokyo, Japan). Neusilin^®^ grade US2 was obtained from Fuji Chemical Co., (Toyama, Japan). 

### 2.3. Analysis of Curcumin (CUR) and Lansoprazole (LNS) Using Ultra-High Performance Liquid Chromatography (UHPLC)

The current analytical study was conducted to simultaneously analyze the model drugs curcumin and lansoprazole in one simple run. Therefore, the aim was to develop a simple, precise and fast method and validate the method to quantify both drugs in the lipid-based SNEDDS formulation and marketed product using UHPLC.

#### 2.3.1. UHPLC Chromatographic Conditions

The study employed a highly sensitive UHPLC system that consisted of a Dionex^®^ UHPLC binary solvent manager, a Dionex^®^ automatic sample manager and a Photodiode Array (PDA) eλ detector which is procured from Thermo scientific, Bedford, MA, USA. The mobile phase was an isocratic mixer of acetonitrile and ammonium formate (pH 2.5) at 40/60% *v*/*v* [26]. The mobile phase was freshly prepared, thereby filtered through an online 0.20 µm filter and degassed constantly by an online degasser within the UHPLC system. The flow rate of the mobile phase was 0.3 mL/min. A kinetex^®^, Phenomenex UPLC C18 column (2.1 × 50 mm, 1.6 µm) maintained at 45 °C was used for the analysis. The total run time was 4 min. The detector wavelengths were set at 285 nm and 428 nm for LNS and CUR, respectively. The injection volume was 1 µL. 

#### 2.3.2. Linearity and Calibration

Appropriate volume of LNS and CUR stock solution (1000 µg/mL) was utilized in the preparation of seven non-zero standard drug concentrations covering the calibration range of 0.1–50.0 ppm. Each standard solution from 0.1 ppm to 50.0 ppm has been injected as six replicates every day on three successive days for validation. The linearity of the results were statistically calculated by employing linear regression equation and correlation coefficient (R2) [31].

#### 2.3.3. Accuracy and Precision

The intra-day accuracy and precision of the proposed method were evaluated by analyzing six replicates of LNS and CUR standards (0.5, 1, 5, 10, 25, 50 ppm) within the same day. Similarly, the inter-day accuracy and precision were also obtained during the three consecutive days using six replicates analysis of the same number of the samples. The complete precision and accuracy of the method was specified as relative standard deviation (RSD) and as % drug recovered, respectively [32].

#### 2.3.4. Limit of Detection (LOD) and Lower Limit of Quantification (LLOQ)

The detection and quantification levels were resolved by sequential dilutions of LNS and CUR stock solutions in order to obtain a signal to noise (S/N) ratio of at least ≈ 3:1 for LOD and ≈ 10:1 for LLOQ [32].

### 2.4. Self-Nanoemulsifying Drug Delivery Systems (SNEDDS) Development and Characterization

#### 2.4.1. Preparation and Drug Loading

Liquid SNEDDS were prepared initially using naturally obtained long chain fatty acids (C16–20) with medium chain mono and diglycerides (C10–12) with 50% non-ionic surfactant. The produced mixture was efficiently homogenized then the model drugs were separately loaded in the liquid SNEDDS with continuous homogenization until the drug completely dissolved. The two lipid-based formulations were experimented on using two bioactive oils namely; black seed oil (BSO), zanthoxylum rhetsa oil (ZRO), a co-surfactant; Imwitor 988 (I988), and a non-ionic surfactant; Kolliphor EL (KrEL), (Table 1) [26].

#### 2.4.2. Formulation Assessment and Characterization

Each formulation was diluted in distilled water at (1:1000 *w*/*w*) ratio and subsequently evaluated for their appearance, homogeneity and spontaneity [33]. The mean droplet size and polydispersity index (PDI) of the (1:1000 *w*/*w*) diluted formulations were measured by photon correlation spectroscopy (PCS) using a Zetasizer Nano ZS analyser (Model ZEN3600, Malvern Instruments Co., Worcester- 200 shire, UK). The particle size of the aqueous dispersions was evaluated using dynamic light scattering (DLS) mode at 25 °C. Zeta potential of each formulation was evaluated by laser Doppler velocimetry (LDV) mode at 25 °C. The average particle size, polydispersity index and zeta potential were determined by taking the mean of three replicates [33,34]. 

The loading capacity of CUR/LNS within the SNEDDS was determined using a simple shake flask method explained by Mohsin et al. previously [35]. The dissolved drug was analyzed by using UHPLC. 

### 2.5. Solidification of CUR and LNS Loaded Liquid SNEDDS Using Adsorbent Neusilin^®^ US2

#### 2.5.1. Curing Process of the Adsorbent Neusilin^®^ US2

Commercially available silicates are mesoporous with small pore sizes of (1 to 50 nm) which usually lead to incomplete emulsification of SNEDDS due to their small size pores and thus incomplete drug release. Therefore, NUS2 powder was cured by a solvent evaporation technique. 

Initially, 100 mg of PVP K-30 (polyvinylpyrrolidone) was weighed and dissolved in 10 mL of the organic solvent then 1 g of the silica was added to the solution which resulted in slurry-like solution. It is critical to select the proper solvent that only dissolve the hydrophilic polymer (PVP), and do not dissolve the silica, to avoid altering the particle size and micrometric properties of the adsorbent. The solvent was then evaporated by keeping the slurry open in the fume hood for 48 h [36,37].

#### 2.5.2. Brunauer–Emmett–Teller (BET) Surface Area of Cured/Uncured Adsorbent

The BET surface area, pore volume and pore size of the cured/uncured NUS2 samples were analyzed by using Micromeritics (Gemini VII, 2390 Surface Area and Porosity USA). The sample of about 0.012–0.034 g was degassed at 80 °C (under N2 flow) for 2 h for moisture and volatile gasses removal before analysis. The adsorption and desorption isotherm at standard temperature and pressure (STP) was obtained in the range of relative pressure from 0.0 to 0.1 [38].

#### 2.5.3. Preparation of Solid SNEDDS Using Uncured/Cured NUS2

Neusilin^®^ grade US2 (aluminum metasilicate, inorganic material) was used as microporous inorganic adsorbent to load all the liquid SNEDDS. Adsorbent Neusilin^®^ US2 was gradually added to the amount of liquid SNEDDS in the ratio of 1:1 *w*/*w* (Table 1). Then, the mixture was thoroughly mixed until uniform solid powder was achieved [26]. The prepared solidified SNEDDS were stored in airtight glass vials for further use.

### 2.6. Characterization of Solid SNEDDS

#### 2.6.1. Scanning Electron Microscopy (SEM) Powder SNEDDS

The solid powder samples (pure LNS, pure CUR, CUR loaded S-SNEDDS and LNS-loaded S-SNEDDS) were examined using scanning electron microscope (Carl Zeiss EVO LS10; Cambridge, UK) to evaluate the effect of solidification on the adsorbent particle shape and detect the signs of incomplete solidification. Samples were fixed on stubs using double-sided adhesive carbon tape then coated with gold in a Q150R sputter coater unit (Quorum Technologies Ltd., East Sussex, UK) under vacuum for 60 s in an argon atmosphere (20 mA) [26].

#### 2.6.2. Differential Scanning Calorimetry (DSC)

The solid powder samples (pure LNS, pure CUR, CUR loaded S-SNEDDS and LNS-loaded S-SNEDDS) were analyzed using a DSC-60 Shimadzu instrument (Kyoto, Japan). Samples (~7 mg) was weighed in a non-hermetically sealed aluminum pan. The samples were heated from 50 to 250 °C at a heating rate of 10 °C/min. The measurements were carried out in nitrogen atmosphere at 40 mL/min flow rate [39].

#### 2.6.3. X-ray Diffraction (XRD)

The solid powder samples (pure LNS, pure CUR, CUR loaded SNEDDS and LNS-loaded S-SNEDDS) were examined by Ultima IV diffractometer (Rigaku, Japan) over the 3–140° 2θ range at a scan speed of 0.5 deg./min by following the previously published method [39].

### 2.7. Fourier Transform Infrared Spectroscopy (FTIR)

FTIR studies were performed to examine whether any possible interaction is existing among the drugs LNS and CUR and lipid formulations. The chemical properties and complexation of powdered samples was performed by Fourier transform infrared spectroscopy (FTIR Spectrum BX from Perkin Elmer LLC., Hopkinton, MA, USA). Pure LNS, pure CUR and LNS/CUR solid SNEDDS powders were compressed for 5 min at 5 bars on a KBr press and the spectra were scanned on the wave-number range of 400–4000 cm^−1^ [26].

### 2.8. Filling of Solid CUR-SNEDDS into Capsules

Solid LNS-SNEDDS powder was filled in enteric coated hard gelatin capsules (Cap A) and solid CUR-SNEDDS was filled in HPMC hard gelatin capsules (Cap B). In this way, Capsule B containing CUR would dissolve and release CUR in gastric media (at pH 1.2) and Cap A containing LNS would dissolve and release LNZ only in intestinal media (at pH 6.8). This approach was expected to be very beneficial in enhancing LNS and CUR stability while maintaining the solubilization benefits of solid SNEDDS. 

### 2.9. In Vitro Dissolution Studies

The experiment involved investigating pure drug powder (LNS and CUR pure powder) and drug-loaded solidified SNEDDS (SF-C and SF-UC). The first experiment involved the dissolution medium comprising 500 mL of simulated gastric fluid (SGF, pH 1.2, 0.1 N HCl with no enzymes) equilibrated at 37 °C. Few turns of non-reactive wire-helix were attached to each capsule to prevent its floating [40]. The dissolution studies were conducted using USP dissolution apparatus II (UDT-804, LOGAN Inst. Corp., USA) with a paddle stirrer being maintained at 50 rpm. Samples of 2 mL were withdrawn at predetermined time intervals 5, 10, 15, 30, and 60 min through 10 micron filter tips (LOGAN Instruments Corp., Somerset, NJ, USA). Samples were centrifuged for 5 min at 9800 g then an aliquot of the supernatant was analyzed using the adopted UHPLC^®^ method. After completing the first phase of running samples (1 h), the pH of the dissolution medium was shifted to 6.8 to simulate the intestinal pH. This was achieved by addition of 250 mL of 0.3 M dibasic sodium phosphate to the media. In this media (pH 6.8), then samples were collected at the same time intervals, centrifuged and assayed as before [40].

LNS-loaded solid SNEDDS powder was used to fill enteric-coated hard gelatin capsules. Therefore, LNS dissolution experiment was directly carried out in 500 mL of simulated intestinal fluid (SIF, phosphate buffer at pH 6.8 with no enzymes). In this experiment, samples were withdrawn at 5, 10, 15, 30, 60 and 120 min and treated as before. The dissolution efficiency (DE)% was utilized to evaluate the drug release from different formulations [41].

### 2.10. Accelerated Stability Study

The stability of CUR in liquid and solid SNEDDS were studied at accelerated condition. Both liquid and solid SNEDDS (filled in air-tight amber glass vials) were stored at 40 ± 2 °C and relative humidity (RH) of 75 ± 5% in climatic stability chambers (Model VC 0100, Vötsch Industrietechnik GmbH, Balingen, Germany) located in Al-Jazeera Pharmaceutical Industries Company, Riyadh, KSA [42,43]. Samples were withdrawn after a minimum of 3 and 6 months, then allowed to room temperature prior to investigation. 

### 2.11. Statistical Analysis

IBM SPSS Statistics 26 software was utilized to analyze the data. One-way analysis of variance (ANOVA) followed by post hoc tests (LSD) were used to compare the dissolution results. A paired *t*-test was used to evaluate the effect of SNEDDS solidification on drug stability. A value for *p* < 0.05 was considered as significant [26].

## 3. Results

### 3.1. Optimization of UHPLC Peak Separations

**Figure 2:**
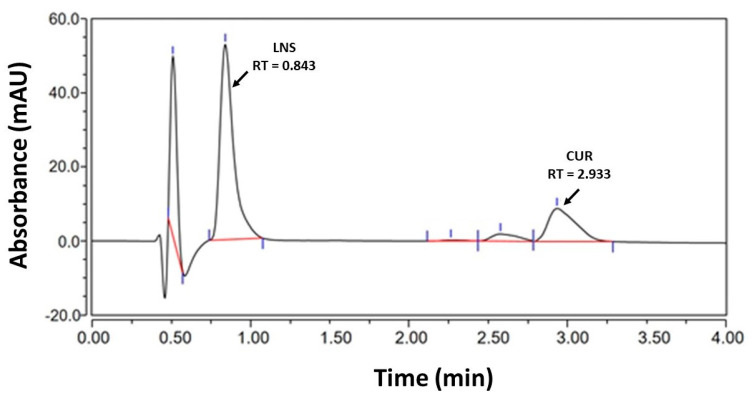
The ultra-high performance liquid chromatography (UHPLC) chromatogram of drug (curcumin (CUR) and lansoprazole (LNS))-loaded lipid-based self-nanoemulsifying drug delivery systems (SNEDDS) formulation.

The developed UHPLC assay showed good separation between LNS and CUR peaks with no interference between them. The chromatographic results of UHPLC technique in the current analysis showed that both LNS and CUR can be accurately quantified in the self-emulsifying lipid formulations (SNEDDS) with high sensitivity and selectivity. The LNS and CUR analytes were well separated at retention time of ≈0.843 min and ≈2.933 min, respectively, with no interference of mobile phase or formulation excipients (Figure 2). The total chromatographic run time was ≈4 min, where the LNS and CUR peaks were of good shape and completely resolved.

#### 3.1.1. Linearity and Calibration

**Figure 3:**
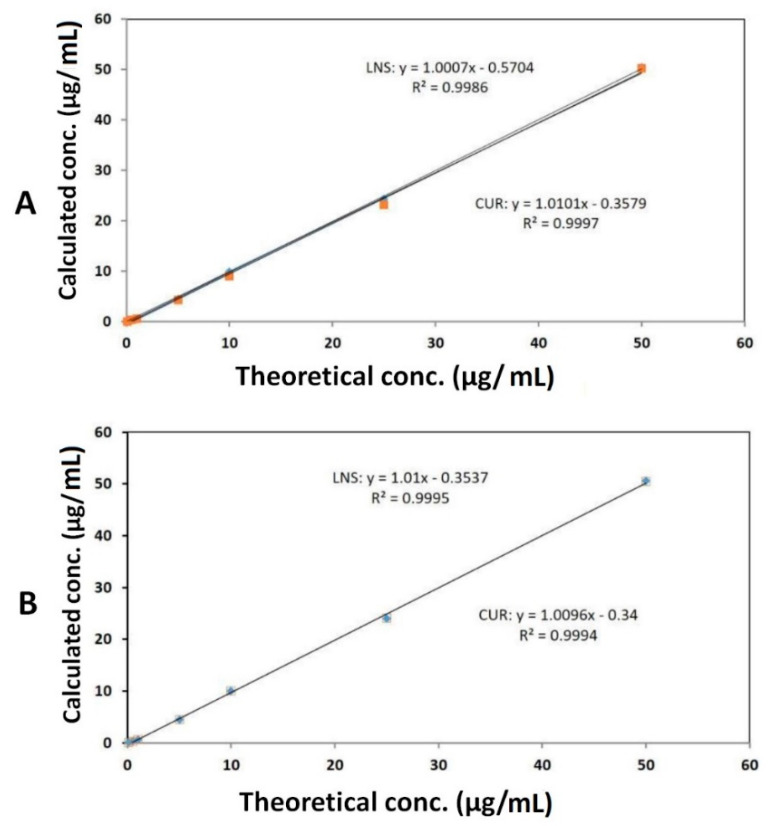
Calibration curves of CUR and LNS in standard solution from (**A**) intra-day and (**B**) inter-day analysis.

The peak responses of both CUR and LNS were linear over the concentration range between 0.10 and 50 μg/mL. Under the above described experimental conditions, the calibration curve of chromatographic peak area versus CUR and LNS concentrations have shown good linear dynamic range in both intra-day and inter-day analyses. These results showed an excellent linear method over the interval studied with correlation coefficient, CUR (r²) = 0.9997 and 0.9994 for intra and inter-day analysis, respectively. While LNS (r² = 0.9986 and 0.9995 for intra and inter-day analysis, respectively (Figure 3).

#### 3.1.2. Accuracy and Precision

**Intra-day:** Within the analytical concentration range of 0.5–50.0 ppm, the RSD% values ranged from 0.2–7.9% for CUR and 0.2–11.5% for LNS (Table 2). The % recovery values ranged from 89.4–101.0% for CUR and 93.0–100.7 for LNS (Table 2). 

**Inter-day:** The % recovery values ranged from 92.7–100.6% for CUR and 90.6–101.1% for LNS (Table 2). While, the RSD% values ranged from 0.3–3.4% for CUR and 0.1–9.6% for LNS (Table 2).

Therefore, the current method showed low RSD% values (≤15%) and the percentage recoveries were within ±15% of nominal concentrations thus the current analytical method met the FDA acceptance criteria for accuracy and precision [44]. 

#### 3.1.3. Limit of Detection and Quantification

LOD and LOQ of CUR and LNS of the proposed method were estimated using the signal-to-noise ratio of 3 for determining LOD and 10 for determining LOQ. The LOD of CUR was found to be 41 ng/mL and 29 ng/mL for LNS at a signal-to-noise ratio of 3. On the other hand, the LOQ were found to be 83 ng/mL for CUR and 45 ng/mL for LNS at a signal-to-noise ratio of 10, respectively.

#### 3.1.4. Method Application and Matrix Effect: Determination of CUR and LNS in Marketed Product of Future-Biotics^®^ and Ultrazole^®^

In order to apply our current UHPLC simultaneous method to assess CUR and LNS, the commercial product of curcumin (Future-Biotics^®^) and lansoprazole (Ultrazole^®^) were purchased individually due to the unavailability of a combined dose in the market. The sample preparation procedure was carried out separately using the suitable solvent. Six replicate determinations for CUR and LNS were performed. Satisfactory results were obtained in good agreement with the label claimed. The % of the labeled claim of CUR and LNS commercial products were found to be 98.8% and 96.5%, respectively (Table 3). The UHPLC chromatograms of CUR and LNS (presented above) were matched with the same retention time of both drugs.

### 3.2. Liquid SNEDDS Development and Performance

The results from formulation characterization showed that F2: BSO:I988 (7:3)/KrEL (1:1) systems showed more transparent appearance, significantly lower droplet size and PDI compared to F1: ZRO:I988 (7:3)/KrEL [1:1] (Table 4). On the other hand, both formulations showed comparable zeta potential values that ranged from −19 to −21 mV. 

Amongst the two anhydrous formulations, F1 (ZRO: I988 (7:3) with KrEL at ratio [1:1]) provided 37.8 mg/g solubility of CUR and 13.3 mg/g LNS with clear appearance upon aqueous dilution. Formulation (F2), which contained the same surfactant but different oil (BSO) showed the comparably lower solubility (23.2 mg for CUR and 10.2 mg for LNS) in anhydrous formulations (Table 4). 

### 3.3. Solidification of Liquid SNEDDS on Neusilin US2

#### 3.3.1. Physical Appearance

**Figure 4:**
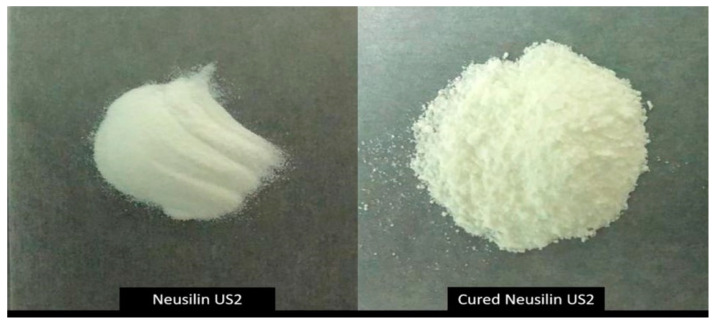
The inorganic silica material Neusilin US2 as uncured (purchased from the manufacturer) and cured (processed) form.

No significant change was observed in the adsorbent physical appearance or flowability after the curing process (Figure 4). In addition, the solidified SNEDDS using cured and uncured NUS2 showed complete solidification with no particle agglomeration or residual oily excipients. 

#### 3.3.2. Brunauer–Emmett–Teller (BET) Surface Area

**Figure 5:**
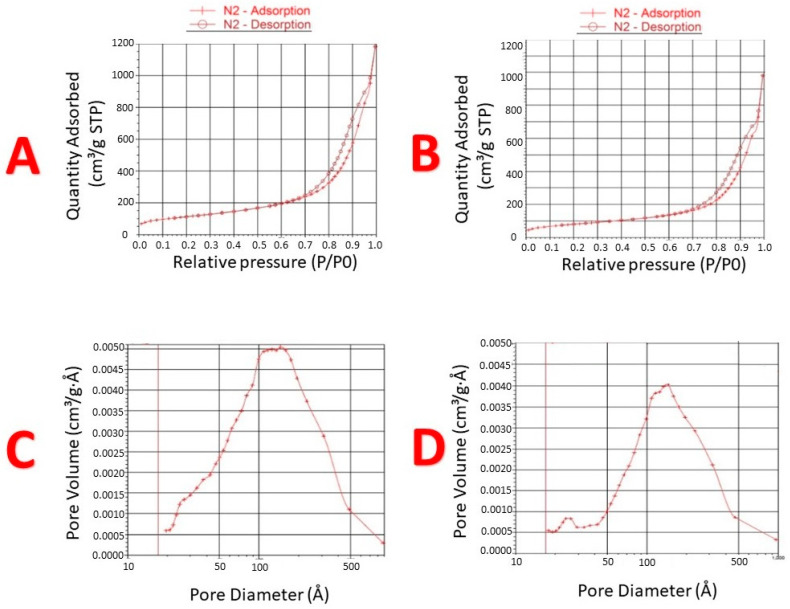
Brunauer–Emmett–Teller (BET) surface area and pore size/volume of cured and uncured NUS2. (**A**,**B**) represents Isotherms linear plot of uncured and cured Neusilin US2 samples; (**C**,**D**): BJH adsorption dV/dD Pore Volume of uncured and cured NUS, respectively.

The BET study revealed significant reduction of BET surface area and pore volume in cured NUS2 samples compared to uncured NUS2 (Table 5). Both cured and uncured NUS2 manifested a Type V adsorption isotherm where cured NUS2 showed ~17% reduction in quantity adsorbed compared to uncured NUS2 (Figure 5A,B). Alternatively, the average pore size was slightly increased from 18.3 nm (uncured NUS2) to 21.2 nm (cured NUS2) (Table 5, Figure 5C,D).

### 3.4. Characterization of Solid SNEDDS

#### 3.4.1. Scanning Electron Microscopy (SEM)

**Figure 6:**
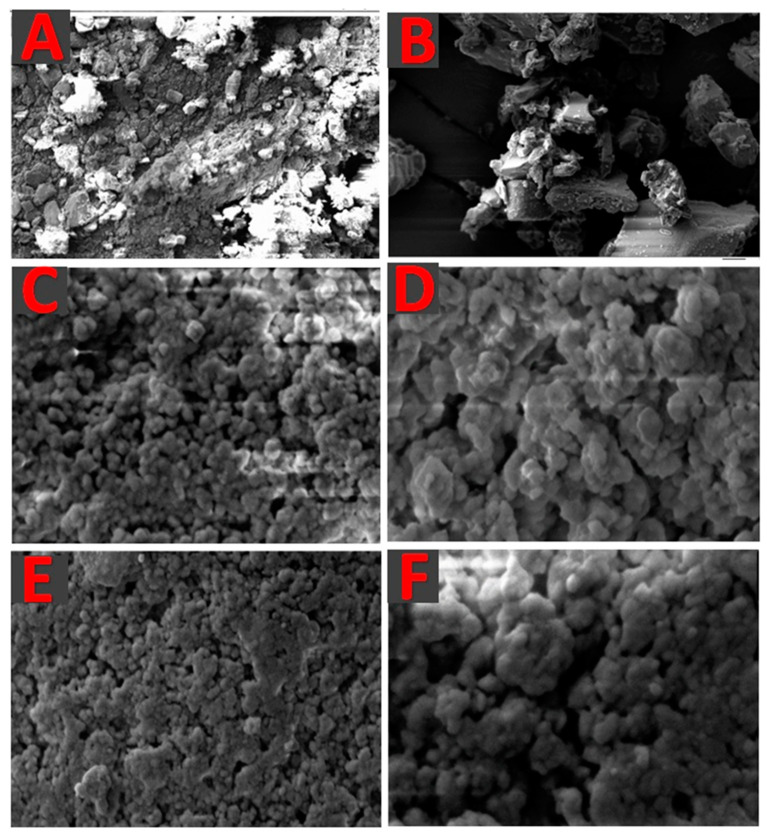
Scanning electron microscopy images of (**A**) pure CUR, (**B**) pure LNS, (**C**) CUR loaded SF1-C, (**D**) LNS loaded SF1-C, (**E**) CUR loaded SF1-UC, and (**F**) LNS loaded SF1-UC, respectively.

SEM images of pure CUR, pure LNS, and representative CUR/LNS-loaded S-SNEDDS are presented in Figure 6. The images illustrated the arrangement of pure CUR (control), pure LNS (control) and the drug loaded solid formulations with Neusilin US2. Pure CUR and LNS (control) were observed to be irregular in shape while CUR and LNS–SNEDDS were observed to be relatively spherical and regular in shape. Most importantly, CUR and LNS SNEDDS were completely solidified with no signs of residual oily excipients. This finding was also confirmed from the texture and physical appearance of the solidified sample. 

#### 3.4.2. Differential Scanning Calorimetry (DSC)

**Figure 7:**
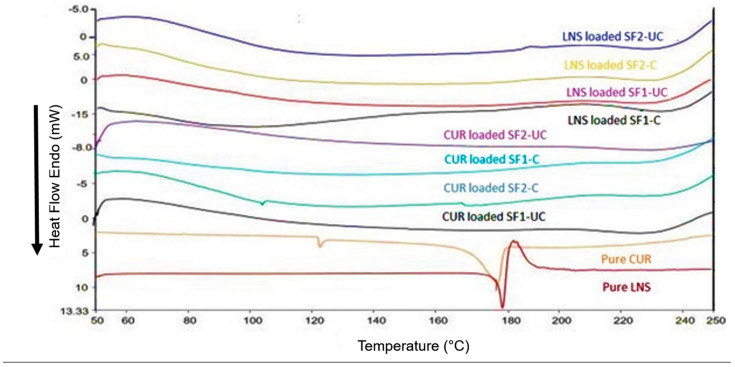
Differential scanning calorimetry (DSC) thermograms of pure CUR/LNS powder and CUR/LNS-loaded solid SNEDDS and SF2-C, respectively. The exact compositions of each formulation are presented in Table 1.

Pure CUR) showed a single sharp endothermic melting peak at 176 °C (corresponding to the drug melting point). While, pure LNS showed a sharp endothermic peak at 178 °C (corresponding to the melting point) followed by an exotherm (corresponding to decomposition process). DSC chromatograms of CUR and LNS-loaded solid SNEDDS did not contain such characteristic endothermic peaks in either uncured or cured NUS2-based samples (Figure 7).

#### 3.4.3. X-ray Powder Diffraction (XRD)

**Figure 8:**
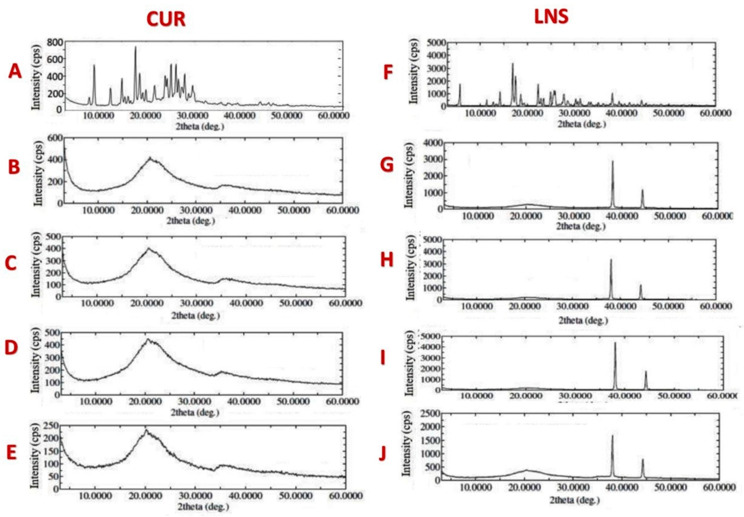
X-ray powder diffraction of (**A**) pure CUR, (**B**,**C**) CUR loaded SF1-UC & SF1-C, (**D**,**E**) CUR-loaded SF2-UC and SF2-C SNEDDS, (**F**) pure LNS, (**G**,**H**) LNS loaded SF1-UC and SF1-C, (**I**,**J**) CUR loaded SF2-UC and SF2-C.

Both Pure CUR and pure LNS manifested the distinct peaks particularly at 2θ: 3–30° indicating the highly crystalline nature of the drugs (Figure 8A,F). The XRD pattern of CUR-loaded solid SNEDDS showed broad peaks at about 22.3° and 36.5° which might be due to the presence of two or more amorphous compounds, with different diffraction patterns, in the solid SNEDDS sample [45]. While, LNS-loaded solid SNEDDS showed broad peaks at 20°. 

#### 3.4.4. Fourier Transform Infrared Spectroscopy (FTIR)

**Figure 9:**
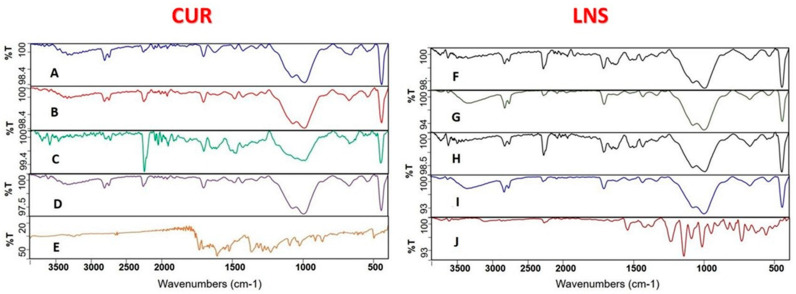
FTIR spectra of CUR/LNS solid SNEDDS. The formulations represent (**A**): CUR loaded SF1-C; (**B**): CUR loaded SF1-UC; (**C**): CUR loaded SF2-C; (**D**): CUR loaded SF2-UC; (**E**): Pure CUR powder; (**F**) LNS loaded SF1-C; (**G**): LNS loaded SF1-UC; (**H**): LNS loaded SF2-C; (**I**): LNS loaded SF2-UC; (**J**): Pure LNS powder.

The current CUR and LNS formulations were intended to be filled into different capsules (using capsule-in-capsule technique). Therefore, no interaction between the two drugs was anticipated and the FTIR samples were prepared by loading either CUR or LNS alone in each formulation. Interestingly, the FTIR showed identical spectra for CUR-loaded and LNS-loaded SNEDDS formulations, providing that the formulation composition is the same (Figure 9A/F,B/G,C/H,D/I). In addition, no significant change of FTIR spectra was observed between cured and uncured solid CUR/LNS SNEDDS.

### 3.5. In Vitro Dissolution of Curcumin and Lansoprazole

**Figure 10:**
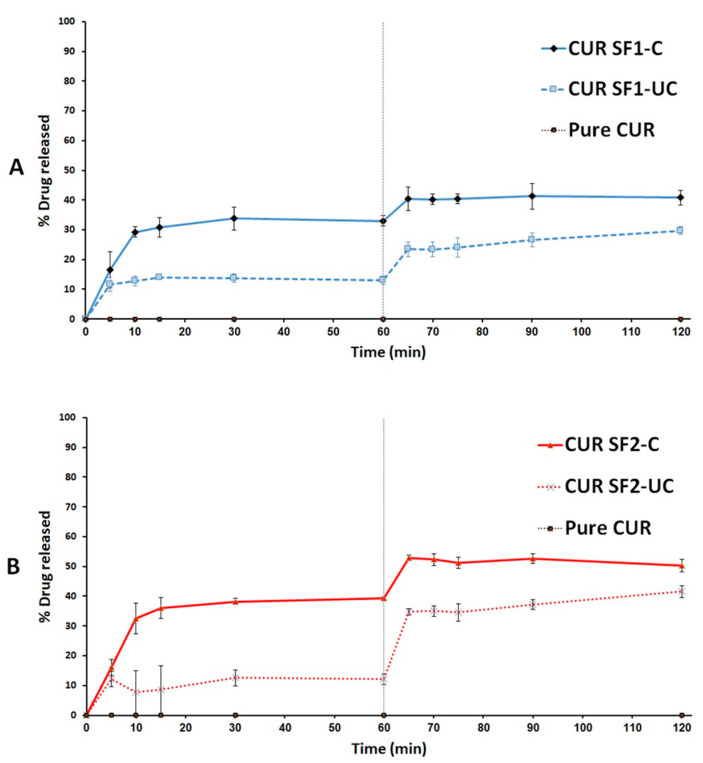
In vitro dissolution profiles of (**A**) ZRO-based and (**B**) BSO-based CUR-loaded solid SNEDDS. Dissolution was carried out in simulated gastric (pH 1.2) for 1 hr and subsequently shifted into simulated intestinal (pH 6.8) media for another 1 h. Pure CUR dissolution data are adopted from [26]. CUR solid SNEDDS data are expressed as mean ± SD, *n* = 3.

Solid SNEDDS (using cured and uncured NUS2) showed significant enhancement of CUR release as follows: SF1-UC (using uncured NUS2) could release ~30% CUR during 120 min dissolution time which was further improved to 41% for SF1-C (using cured NUS2) (Figure 10). Similarly, the CUR release was improved from ~41% to >50% upon switching from SF2-UC to SF2-C (Figure 10). 

**Figure 11:**
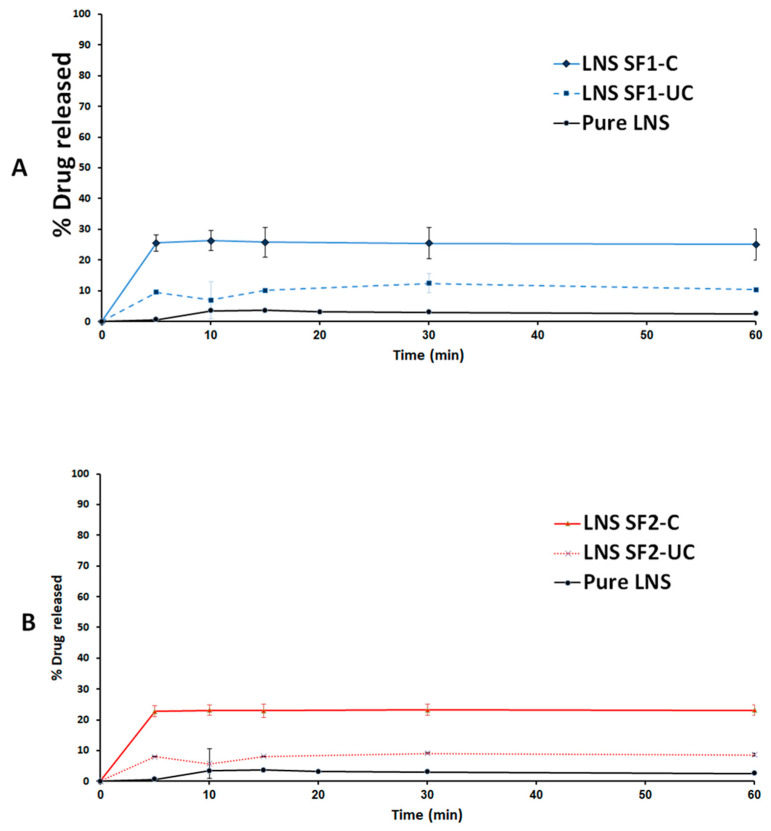
In vitro dissolution profiles of (**A**) ZRO-based and (**B**) BSO-based LNS-loaded solid SNEDDS. Dissolution was carried out in simulated intestinal (pH 6.8) media for 1 h. Pure LNS was used as a control. LNS solid SNEDDS data are expressed as mean ± SD, *n* = 3.

Because LNS formulation was filled into enteric coated capsules, LNS dissolution was presented at pH 6.8 only. The pure LNS powder was released as low as 3% during the dissolution studies until 60 mins in neutral media (pH 6.8). Interestingly, solid SNEDDS (using cured and uncured NUS2) showed significant enhancement of LNS release as follows. SF1-UC (using uncured NUS2) could release ~10% CUR during 60 min dissolution time which was further improved to 28% for SF1-C (using cured NUS2). Similarly, LNS release was improved from ~8% to 24% upon switching from SF2-UC to SF2-C (Figure 11). 

### 3.6. Stability at Accelerated Conditions

**Figure 12:**
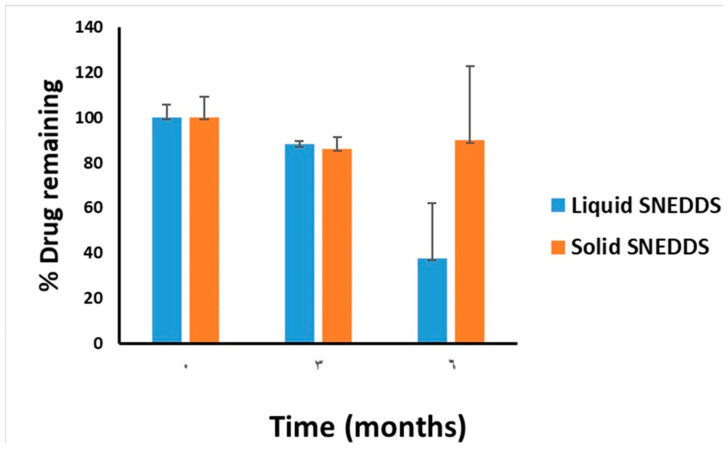
The stability data of liquid and solid SNEDDS at accelerated storage conditions. Data are expressed as % of CUR concentration relative to initial concentration at 0 time. The liquid SNEDDS and solid SNEDDS were represented by F1 and F1-C, respectively.

CUR showed significant degradation in liquid SNEDDS at 3- and 6-month time intervals (Figure 12). At 6 months, the intact CUR amount decreased to 38% of the initial value. In contrast, the solid SNEDDS was able to maintain significantly higher CUR amount (>86%) after 6 months’ storage at accelerated conditions (Figure 12).

## 4. Discussion

Self-nanoemulsifying drug delivery systems (SNEDDS) have remained in the forefront in terms of their ease of preparation, unmatched formulation characteristics, and solubility/bioavailability enhancement. In terms of formulation characterization, the formulation F2-BSO:I988 (7:3)/KrEL [1:1] showed the most transparent appearance, lowest droplet size (14 nm) and lowest PDI (0.1) compared to the other formulation. These findings reveal the excellent self-emulsifying properties of BSO which have been confirmed through its ability to form ultrafine-nanoemulsions with wide range of surfactant and/or cosurfactants as reported recently in several publications [20,25,26]. Upon changing the oil portion from BSO to ZRO, F1- ZR:I988 (7:3)/ KrEL [1:1], the droplet size has significantly increased from 14 to 158 nm, yet it remained in the nanoscale. Overall, the both F1 and F2 -SNEDDS showed superior formulation characteristics in terms of lower droplet size and PDI, hence, were selected for the solidification studies using adsorbent Neusilin US2 (NUS2).

Zeta potential (ZP) is another parameter in assessing SNEDDS formulation. The significance of ZP value could be correlated with formulation stability upon nanoemulsification. In the current study, all the formulations showed acceptable ZP values that ranged from −19 to −21 mV. These results are in agreement with previous studies that reported negative zeta potential values for most SNEDDS formulations [46,47]. 

Adsorption onto high surface area inorganic silica materials has been commonly used to solidify liquid SNEDDS into free-flowing powders. In the current study, all solid SNEDDS presented acceptable free-flowing properties which reflects the proper ratios of the NUS2 adsorbent to liquid SNEDDS (1:1, *w*/*w*). In addition, the adsorbent reserved its flowability and physical appearance after being cured with PVP-k30.

The successful coating of PVP on silica pores could be confirmed by the significant reduction of BET surface area and pore volume in cured NUS2 samples. Interestingly, the average pore size was slightly increased in the cured NUS2 sample which reveals that the coating process was efficient enough to penetrate deeply into the small-sized pore within the silica adsorbent. This fact could be confirmed by analyzing the differential surface area parameters. In uncured NUS2 sample, the micropore area (the smaller-size pores) represented 6% of the total sample surface area where 94% of the area were expressed at the external surface of the adsorbent (the relatively larger pores) (Table 5). On the other hand, cured NUS2 showed a significant (50%) reduction in the micropore area (%). Alternatively, the external surface area of cured NUS2 was increased to 97%. This finding is of great interest to our study scope because blocking very small pores could allow more space for the self-emulsification process to take place in the larger-sized pores.

Thermodynamic techniques are applied for determining the thermal stress of the pure drug and the excipients as well as their interactions during the formulation process. The DSC of pure CUR and pure LNS showed sharp endothermic peaks which correspond to the drugs melting points, indicating their characteristic crystalline nature. The current DSC results of pure CUR and LNS are in strong agreement with previous studies [48,49,50]. Rosenblatt et al. presented a detailed investigation of the thermal behavior of lansoprazole and other related sulfoxides. All the drugs behaved similarly and showed endothermic peaks followed by exotherms corresponding to the drug decomposition [49]. 

Interestingly, the drug characteristic peaks disappeared within all CUR and LNS loaded solid SNEDDS formulations. This suggests the conversion of crystalline CUR and LNS to the amorphous form which could be attributed to complete dissolution of the drug in the solid SNEDDS. This phenomenon was also true for all the CUR and LNS-loaded solid SNEDDS formulations investigated in the current studies.

Pure CUR showed multiple characteristic diffraction peaks with the highest intensity ones within the range 8–30° [50]. In correlation with DSC results, the XRD data showed that the drug-related characteristic peaks significantly disappeared in all CUR-loaded solid-SNEDDS samples, which indicates that CUR were not present in crystalline form within the solid SNEDDS and the amorphous state would contribute to the higher drug-loading capacity of SNEDDS.

In agreement with previous studies, pure LNS showed multiple characteristic diffraction peaks with the highest intensity ones within the range 6–30° [51]. In contrast, LNS-loaded solid SNEDDS samples showed broad diffraction peaks at 20° and the LNS-related high intensity diffraction peaks disappeared except for the two diffraction peaks at 35–45°. These findings, together with the DSC findings, strongly suggest that LNS was converted to an amorphous state within solid SNEDDS [52]. In addition, the significant disappearance of CUR and LNS characteristics peaks in FTIR spectra of all solid SNEDDS samples implies that both drugs were successfully captured inside the solid SNEDDS [53]. The similarity of FTIR spectra, between the corresponding cured/uncured and CUR/LNS solid SNEDDS, suggest that no chemical interaction took place between solid SNEDDS excipients and CUR, LNS or PVP.

As reported in previous studies, pure CUR showed negligible release due to its extreme hydrophobicity and poor aqueous solubility [20,54,55,56]. Solid SNEDDS using uncured NUS2 led to significant inhibition of CUR release compared to cured-NUS counterparts. This finding was consistent for both CUR and LNS and within different SNEDDS formulations (F1 and F2) (Figure 10 and Figure 11). Similar findings of significant release inhibition from uncured inorganic silica adsorbent samples have been reported [20,36]. Several mechanisms could be involved in the phenomenon of the declined drug release extent upon SNEDDS adsorption onto uncured silica as follows: the decreased drug release could be due to gel formation that clogs the meso pores of the silicate, thus trapping the liquid SNEDDS inside [57]. Another explanation could be based on SNEDDS retention within the mesoporous interiors (pore size = 2–50 nm) which do not have sufficient room for emulsification compared to the macro porous structure of the adsorbent (pore size > 50 nm) which provide more physical space for emulsification process [37]. Some studies also proposed that the development of physical bonds between the drug and carrier could favor drug diffusion from the SNEDDS to the surface of the adsorbent followed by drug nucleation and precipitation which in turn hinders complete drug release from the solid SNEDDS [38].

The current study showed that curing NUS2 with 10% PVP led to significant enhancement of CUR and LNS dissolution efficiencies (to 1.82- and 2.75-fold, respectively) compared to uncured NUS2-based solid SNEDDS. Similar findings were reported with cured silica adsorbents [36,38]. These results could be attributed to blocking the very small pores in the micro/mesoporous regions of NUS2 by applying the hydrophilic polymer (PVP k30). This hypothesis could be confirmed by increased average pore size and significant reduction of micropore area% in cured NUS2 samples, in the current study. Accordingly, SNEDDS would not penetrate into such very small-sized pores and, therefore, minimal drug-adsorbent interaction and enhanced drug release could be achieved. Furthermore, the adsorbent hydrophobic surface could be masked by hydrophilic polymer. Therefore, precipitation of drug due to adverse interaction between drug and silica could also be prevented [38]. In addition, the pre-coated hydrophilic polymer could facilitate water penetration into the silicate by wicking action and, hence, facilitate drug release [36].

Previous studies showed that CUR is more structurally stable in acidic environments compared to neutral and alkaline [58]. Interestingly, previous studies suggested that CUR chemical stability can be improved by encapsulation with lipids or nanoparticles [59]. These data are in good correlation with the current study results. CUR lipid-based solid SNEDDS showed enhanced CUR release at neutral media (pH 6.8) up to 1 h (Figure 10) which was suggested to be owing to CUR partitioning inside the lipid-based nanoemulsion droplets that enhanced aqueous solubility and stability compared to the reported rapid (<10 min) degradation of pure CUR at relative neutral pH values [58].

On the other hand, previous studies reported that lansoprazole is rapidly degraded at acidic environments and, therefore, it must be given in an enteric coated dosage form [60,61]. In the current study, cured solid SNEDDS showed fast and enhanced LNS release compared to pure drug and uncured counterparts at pH 6.8. However, a maximum of 30% release was observed with all solid SNEDDS. Similar findings have been reported with fenofibrate-SNEDDS adsorbed onto cured silica carriers and could be attributed to the non-sink dissolution conditions and/or the partial retention of drug-loaded SNEDDS within the mesoporous interiors of the adsorbent, even after the curing process [38].

Solid SNEDDS showed significant enhancement of CUR stability in formulation compared to liquid SNEDDS. These results are in agreement with previously published work and could be attributed to the fact that chemical reactions (including drug degradation) occur at slower rates in solid state compared to liquid counterparts [42,43,62].

However, in spite of the significant enhancement of drug release observed upon curing NUS2 with 10% PVP-K30, further studies demand to evaluate the impact of using higher proportions of PVP-K30 (>15%) on drug release from solid SNEDDS. Other hydrophilic polymers such as polyvinyl alcohol, HPMC, and Kollidon VA6 could be also explored. In addition, more green solvents’ utilization should be emphasized in curing silica materials to maintain environmentally benign manufacturing conditions and higher product safety attributes.

The current study aimed to design a combined CUR and LNS dosage form using a capsule-in-capsule technique where LNS-SNEDDS-filled Cap A would be inserted into Cap B containing CUR-SNEDDS. In fact, this technique led to some challenges including limited capsule capacity (size) due to placing the small capsule inside the larger one. In addition, the prepared solid SNEDDS powders experienced low bulk densities and, hence, it was difficult to load the CUR and LNS formulations in a single capsule-in-capsule. Future studies might evaluate the feasibility of loading CUR and LNS solid SNEDDS in bi-layer compressed tablets. The excellent flowability and compressibility of solid SNEDDS powder could be potentially useful for tablet manufacturing. A pharmacokinetic assessment would also be valuable to see how much drugs from the dosage forms are available in the systemic circulation, which might help to establish better in vitro and in vivo correlations.

## 5. Conclusions

The representative liquid SNEDDS loaded with antiulcer compounds, CUR and LNS were successfully solidified and encapsulated using hard gelatin capsules. The results from the in vitro dissolution studies demonstrated that both the optimized SF-UC and SF-C were able to show higher a percentage cumulative release as compared to the pure drugs. However, cured NUS2 showed superior drug release compared to the uncured counterpart for both drugs and upon using two different SNEDDS formulations. Solid SNEDDS showed enhanced drug stability compared to liquid SNEDDS with no significant changes observed in physical appearance. The present study suggests that the developed combined oral dosages of CUR and LNS (combination therapy) could be used as a potential product using a lipid-based solid nanocarrier system to deliver a higher amount of curcumin and lansoprazole to the systemic circulation with enhanced antiulcer activity.

## Figures and Tables

**Table 1:** The composition of lipid-based formulation systems developed in the study.

Formulation Code	ZRO (%)	I988 (%)	BSO (%)	KrEL (%)	NUS2 (%)	PVP-K30 (%)
**F1**	35	15	--	50	--	--
**F2**	--	15	35	50	--	--
**SF1-UC**	17.5	7.5	-	25	50	--
**SF2-UC**	--	7.5	17.5	25	50	--
**SF1-C**	17.5	7.5	--	25	45.5	4.5
**SF2-C**	--	7.5	17.5	25	45.5	4.5

The excipients amounts are expressed as weight percentage. ZRO: Zanthoxylum rhetsa seed oil; I988: Imwitor 988; BSO: Black seed oil; KrEL: Kolliphor EL; NUS2: Neusilin US2 SF-UC: solidified formulation using uncured adsorbent; SF-C: solidified formulation using cured adsorbent.

**Table 2:** UHPLC data of intra-day and inter-day accuracy (recovery) and precision (RSD%) of CUR and LNS standards.

Assay Type	Nominal Drug Concentration (ug/mL)	CUR		LNS	
		Accuracy (%)	RSD (%)	Accuracy (%)	RSD (%)
**Intra-Day**	0.5	89.4	7.9	99.0	11.5
	1	91.1	2.9	93.0	0.8
	5	98.8	0.9	98.6	0.6
	10	99.9	0.6	99.8	0.2
	25	98.5	2.0	98.4	2.7
	50	101.0	0.2	100.7	0.4
**Inter-Day**	0.5	92.7	2.6	90.6	9.6
	1	92.9	0.9	92.7	0.4
	5	98.4	0.4	98.6	0.7
	10	99.7	0.3	100.0	0.5
	25	94.8	3.4	96.1	0.3
	50	100.6	0.3	101.1	0.1

**Table 3:** Determination and % recovery of CUR and LNS commercial products.

Real Sample	Manufacturer	Claimed Amount (mg)	Actual Amount (mg) *	% of Labelled Claim
Turmeric Capsules	Futurebiotics^®^, Hauppauge, NY 11788, USA	500	494.15 ± 3.58	98.83
Ultrazole	Riyadh Pharma, Saudi Arabia	30	28.95 ± 2.11	96.50

* Data are expressed as mean ± SD, *n* = 6.

**Table 4:** Droplet sizes, polydispersity index (PDI), zeta potential and solubility parameters (of representative formulations.

Formulation Code	Compositions	Droplet Size (nm)	PDI	Zeta Potential (mV)	SNEDDS	Calculated Solubility (mg/g)	
						CUR	LNS
F1	ZRO:I988 (7:3)/KrEL [1:1]	158.1 ± 18	0.44 ± 0.05	−19.2 ± 1.2	√ Hazy	37.8 ± 3.5	13.3 ± 0.9
F2	BSO:I988 (7:3)/KrEL [1:1]	13.8 ± 0.2	0.12 ± 0.02	−21.3 ± 0.6	√ Transparent	23.2 ± 2.3	10.2 ± 0.3

**Table 5:** Brunauer–Emmett–Teller (BET) surface area of uncured and cured NUS2 samples.

Parameter	Uncured NUS2	Cured NUS2
**BET Surface Area (m²/g)**	399.2	286.4
Micropore area (%) *	6%	3%
External surface area (%) *	94%	97%
Pore volume (cm³/g) **	1.82	1.50
Pore size (nm) ***	18.3	21.2

* Micropore and external surface area were expressed as % out of total BET surface area. ** Calculated as BJH Adsorption cumulative volume of pores between 17.000 Å and 3000.000 Å diameter. *** Calculated as adsorption average pore width (4 V/A by BET).

## Data Availability

Not applicable.
